# Supplementary material for: Social exclusion and the perspectives of health care providers on migrants in Gauteng public health facilities, South Africa
Source: PLoS One. 2020 Dec 28;15(12):e0244080. doi: 10.1371/journal.pone.0244080 (PMC7769451; doi:10.1371/journal.pone.0244080)
Supplement: S1 Questionnaire — (DOCX) [file pone.0244080.s001.docx]

**Healthcare provider questionnaire**

**Thank you for taking the time to participate in this study. Your participation is invaluable!**

Having read and understood the information sheet, do you agree to participate in the study? No/ Yes

Date of field visit: __________________________________

**Section 1: Background characteristics**

How old are you? (in completed years): __________________________________

What is your sex? Male/ Female

Category of health professional:

- Professional nurse
- Enrolled nurse
- Nursing assistant
- Doctor
- Other
- If other, please specify __________________________________

Health facility:

- Central hospital
- Clinic
- Community health centre
- District hospital
- Regional hospital
- Regional Tertiary hospital
- Specialised hospital

How many years have you been working at this facility? __________________________________

The country of your birth? __________________________________

What is your current marital status?

- Married
- Living together
- Single
- Divorced/ Separated
- Widowed

**Section 2: Social exclusionary views or practices among health care providers**

**Part I**

1. In this past year, have you seen incidents of discrimination against migrants and/or refugees in

this facility? No/ Yes

1. In your opinion, are migrants and refugees treated differently in this facility compared to South Africans? No/ Yes

**Part II**

1. Listed below are questions on migrants and refugees. Please specify how strongly you would agree or disagree with each statement by indicating the corresponding number.
2. I am sensitive to the healthcare need of migrants/refugees.

- Strongly disagree
- Disagree
- Disagree slightly
- Neither disagree nor agree
- Agree slightly
- Agree
- Strongly agree

1. I believe migrant and refugee patients should go back to their home country for healthcare.

- Strongly disagree
- Disagree
- Disagree slightly
- Neither disagree nor agree
- Agree slightly
- Agree
- Strongly agree

1. I provide the same quality of care to migrants/refugees as I do to South Africans.

- Strongly disagree
- Disagree
- Disagree slightly
- Neither disagree nor agree
- Agree slightly
- Agree
- Strongly agree

1. I believe that migrant and refugee patients only come to South Africa for healthcare services.

- Strongly disagree
- Disagree
- Disagree slightly
- Neither disagree nor agree
- Agree slightly
- Agree
- Strongly agree

1. I discriminate against migrant/refugee patients.

- Strongly disagree
- Disagree
- Disagree slightly
- Neither disagree nor agree
- Agree slightly
- Agree
- Strongly agree

1. I believe that migrants/refugees should be covered under the National Health Insurance.

- Strongly disagree
- Disagree
- Disagree slightly
- Neither disagree nor agree
- Agree slightly
- Agree
- Strongly agree

1. I have delayed healthcare to patients because of their migrant or refugee status.

- Strongly disagree
- Disagree
- Disagree slightly
- Neither disagree nor agree
- Agree slightly
- Agree
- Strongly agree

d) Do you have any further comments you wish to make?

**END OF QUESTIONNAIRE**
