# Supplementary material for: Social exclusion and the perspectives of health care providers on migrants in Gauteng public health facilities, South Africa
Source: PLoS One. 2020 Dec 28;15(12):e0244080. doi: 10.1371/journal.pone.0244080 (PMC7769451; doi:10.1371/journal.pone.0244080)
Supplement: Attachment — Submitted filename: PONE-D-19-34029_reviewer.pdf [file pone.0244080.s002.pdf]

PLOS ONE

Social exclusion and perceptions of health care providers on migrants in Gauteng public health facilities, South Africa

--Manuscript Draft--

**Manuscript Number:**	PONE-D-19-34029
**Article Type:**	Research Article
**Full Title:**	Social exclusion and perceptions of health care providers on migrants in Gauteng public health facilities, South Africa
**Short Title:**	Health care provider perceptions on social exclusion and migrants
**Corresponding Author:**	Janine White, MSocSci<br>University of the Witwatersrand Faculty of Health Sciences<br>Johannesburg, Gauteng SOUTH AFRICA
**Keywords:**	health care providers; migrants; social exclusion; public health
**Abstract:**	Background : The draft global action plan on migrant health focuses on achieving universal health coverage (UHC) for all people, regardless of citizenship. In South Africa, the proposed National Health Insurance system is the primary UHC reform. Health workers are central to the achievement of UHC, and their attitudes, behaviour or practices could either advance or constrain UHC for migrants. Using a theory of social exclusion, the aim of this study was to examine the perspectives of health care providers on delivering health services to migrants in public health facilities in the Gauteng Province of South Africa.<br>Methods : We used stratified, random sampling to select 13 public health care facilities in Gauteng Province. On the randomly selected fieldwork days, all health care providers in ambulatory care were invited to complete a self-administered questionnaire. In addition to socio-demographic information, the questionnaire measured discrimination against migrants and social exclusionary views or practices. Stata® was used to analyse the data, weighted by type of facility and health care provider category.<br>Results : We recruited 277 health care providers, with a refusal rate of 10%. The majority of participants were female (77.6%), nurses (51.9%) and had worked for an average of 6.8 years in their facilities. 21.0% of health care providers reported that they had witnessed discrimination against migrants, while 22.6% reported differential treatment of migrant patients. Enrolled nurses and nursing assistants were significantly more likely to agree with social exclusionary views or practices (p<0.001). The predictors of less exclusionary views were health care providers born outside South Africa (p<0.05).<br>Conclusion : Health care providers are central to UHC, inclusive of migrants. Social exclusionary views or practices must be addressed through a multi-pronged approach, including training in culture-sensitivity, ethics and human rights; and advocacy to ensure that health care providers uphold their professional obligations to all patients.
**Order of Authors:**	Janine White, MSocSci
	Duane Blaauw
	Laetitia Rispel
**Additional Information:**	
**Question**	**Response**
**Financial Disclosure**<br><br>Enter a financial disclosure statement that describes the sources of funding for the work included in this submission. Review the submission guidelines for detailed requirements. View published research	The author(s) received no specific funding for this work.

Powered by Editorial Manager® and ProduXion Manager® from Aries Systems Corporation

articles from *PLOS ONE* for specific examples.<br><br>This statement is required for submission and **will appear in the published article** if the submission is accepted. Please make sure it is accurate.<br><br>**Unfunded studies**<br>Enter: *The author(s) received no specific funding for this work.*<br><br>**Funded studies**<br>Enter a statement with the following details:<br>• Initials of the authors who received each award<br>• Grant numbers awarded to each author<br>• The full name of each funder<br>• URL of each funder website<br>• Did the sponsors or funders play any role in the study design, data collection and analysis, decision to publish, or preparation of the manuscript?<br>• **NO** - Include this sentence at the end of your statement: *The funders had no role in study design, data collection and analysis, decision to publish, or preparation of the manuscript.*<br>• **YES** - Specify the role(s) played.<br><br>* typeset	
**Competing Interests**<br><br>Use the instructions below to enter a competing interest statement for this submission. On behalf of all authors, disclose any competing interests that could be perceived to bias this work—acknowledging all financial support and any other relevant financial or non-financial competing interests.<br><br>This statement **will appear in the published article** if the submission is accepted. Please make sure it is accurate. View published research articles from *PLOS ONE* for specific examples.	The authors have declared that no competing interests exist.

**NO authors have competing interests**<br><br>Enter: *The authors have declared that no competing interests exist.*<br><br>**Authors with competing interests**<br><br>Enter competing interest details beginning with this statement:<br><br>*I have read the journal's policy and the authors of this manuscript have the following competing interests: [insert competing interests here]*<br><br>* typeset	
**Ethics Statement**<br><br>Enter an ethics statement for this submission. This statement is required if the study involved:<br><br>• Human participants<br>• Human specimens or tissue<br>• Vertebrate animals or cephalopods<br>• Vertebrate embryos or tissues<br>• Field research<br><br>Write "N/A" if the submission does not require an ethics statement.<br><br>General guidance is provided below. Consult the submission guidelines for detailed instructions. **Make sure that all information entered here is included in the Methods section of the manuscript.**	This research was approved by the Human Ethics Research Committee (MEDICAL) of the University of the Witwatersrand, ethics number: M170988.

Powered by Editorial Manager® and ProduXion Manager® from Aries Systems Corporation

**Format for specific study types**<br><br>**Human Subject Research (involving human participants and/or tissue)**<br>• Give the name of the institutional review board or ethics committee that approved the study<br>• Include the approval number and/or a statement indicating approval of this research<br>• Indicate the form of consent obtained (written/oral) or the reason that consent was not obtained (e.g. the data were analyzed anonymously)<br><br>**Animal Research (involving vertebrate animals, embryos or tissues)**<br>• Provide the name of the Institutional Animal Care and Use Committee (IACUC) or other relevant ethics board that reviewed the study protocol, and indicate whether they approved this research or granted a formal waiver of ethical approval<br>• Include an approval number if one was obtained<br>• If the study involved *non-human primates*, add *additional details* about animal welfare and steps taken to ameliorate suffering<br>• If anesthesia, euthanasia, or any kind of animal sacrifice is part of the study, include briefly which substances and/or methods were applied<br><br>**Field Research**<br><br>Include the following details if this study involves the collection of plant, animal, or other materials from a natural setting:<br>• Field permit number<br>• Name of the institution or relevant body that granted permission	
**Data Availability**<br><br>Authors are required to make all data underlying the findings described fully available, without restriction, and from the time of publication. PLOS allows rare exceptions to address legal and ethical concerns. See the PLOS Data Policy and FAQ for detailed information.	No - some restrictions will apply

Powered by Editorial Manager® and ProduXion Manager® from Aries Systems Corporation

A Data Availability Statement describing where the data can be found is required at submission. Your answers to this question constitute the Data Availability Statement and **will be published in the article**, if accepted.<br><br>**Important:** Stating 'data available on request from the author' is not sufficient. If your data are only available upon request, select 'No' for the first question and explain your exceptional situation in the text box.<br><br>Do the authors confirm that all data underlying the findings described in their manuscript are fully available without restriction?	
**Describe where the data may be found in full sentences. If you are copying our sample text, replace any instances of XXX with the appropriate details.**<br><br>• If the data are **held or will be held in a public repository**, include URLs, accession numbers or DOIs. If this information will only be available after acceptance, indicate this by ticking the box below. For example: *All XXX files are available from the XXX database (accession number(s) XXX, XXX.).*<br>• If the data are all contained **within the manuscript and/or Supporting Information files**, enter the following: *All relevant data are within the manuscript and its Supporting Information files.*<br>• If neither of these applies but you are able to provide **details of access elsewhere**, with or without limitations, please do so. For example:<br><br>*Data cannot be shared publicly because of [XXX]. Data are available from the XXX Institutional Data Access / Ethics Committee (contact via XXX) for researchers who meet the criteria for access to confidential data.*<br><br>*The data underlying the results presented in the study are available from (include the name of the third party*	This data is part of a doctoral study, which will be examined in 2020. The ethics number is M170988. The data, codebook, metadata and study documentation is deposited to "Wiredspace" which is an online repository for PHDS and Masters Thesis , data will be published together with the thesis. The permanent link to the repository item will be available end January 2020, contact. Nina.lewin@wits.ac.za Data Services Library. The data in the submitted research article was collected from health care providers working in public health care facilities in Gauteng Province, South Africa and was captured and is stored in REDCap which runs on the facility of University of Witwatersrand health sciences servers. REDcap contains provisions for complying with HIPPA and offers controlled data access. All health care providers gave their consent for confidential participation before participating in the survey. No personal information was collected. Data access is limited for ethical reasons to controlled access within the Redcap server. The data is available to any Bona fide researchers with appropriate ethics clearance from their institution or on application to the University of Witwatersrand Medical ethics committee. However, all data is available outside of the Redcap server to accredited peer reviewers on a specific peer review access.

Powered by Editorial Manager® and ProduXion Manager® from Aries Systems Corporation

and contact information or URL).<br>• This text is appropriate if the data are owned by a third party and authors do not have permission to share the data.<br><br>* typeset	
Additional data availability information:	Tick here if your circumstances are not covered by the questions above and you need the journal’s help to make your data available.

Powered by Editorial Manager® and ProduXion Manager® from Aries Systems Corporation

Manuscript
Click here to download Manuscript White et al Provider paper.docx

Social exclusion and perceptions of health care providers on migrants in Gauteng public health facilities, South Africa

Janine A. White,[1*] Duane Blaauw,[2] and Laetitia C. Rispel[3]

[1] School of Public Health, Faculty of Health Sciences, University of the Witwatersrand, Johannesburg, South Africa

[2] Centre for Health Policy, School of Public Health, Faculty of Health Sciences, University of the Witwatersrand, Johannesburg, South Africa

[3] Centre for Health Policy & Department of Science and Innovation (DSI)/ /National Research Foundation (NRF) Research Chair, School of Public Health, Faculty of Health Sciences, University of the Witwatersrand, Johannesburg, South Africa

***Corresponding author**

Janine A. White

School of Public Health
Faculty of Health Sciences
University of the Witwatersrand, Johannesburg
27 St Andrew's Road
Parktown
2193
South Africa
Telephone: +27 11 717 2193
Email: Janine.white@wits.ac.za

Abstract

Background: The draft global action plan on migrant health focuses on achieving universal health coverage (UHC) for all people, regardless of citizenship. In South Africa, the proposed National Health Insurance system is the primary UHC reform. Health workers are central to the achievement of UHC, and their attitudes, behaviour or practices could either advance or constrain UHC for migrants. Using a theory of social exclusion, the aim of this study was to examine the perspectives of health care providers on delivering health services to migrants in public health facilities in the Gauteng Province of South Africa.

Methods: We used stratified, random sampling to select 13 public health care facilities in Gauteng Province. On the randomly selected fieldwork days, all health care providers in ambulatory care were invited to complete a self-administered questionnaire. In addition to socio-demographic information, the questionnaire measured discrimination against migrants and social exclusionary views or practices. Stata® was used to analyse the data, weighted by type of facility and health care provider category.

Results: We recruited 277 health care providers, with a refusal rate of 10%. The majority of participants were female (77.6%), nurses (51.9%) and had worked for an average of 6.8 years in their facilities. 21.0% of health care providers reported that they had witnessed discrimination against migrants, while 22.6% reported differential treatment of migrant patients. Enrolled nurses and nursing assistants were significantly more likely to agree with social exclusionary views or practices ($p<0.001$). The predictors of less exclusionary views were health care providers born outside South Africa ($p<0.05$).

Conclusion: Health care providers are central to UHC, inclusive of migrants. Social exclusionary views or practices must be addressed through a multi-pronged approach, including training in culture-sensitivity, ethics and human rights; and advocacy to ensure that health care providers uphold their professional obligations to all patients.

Introduction

This millennium has been marked by mass migration [1], with an estimated 70 million displaced people globally in 2019 [2]. In this paper, migrants refer to people who have moved across an international border away from their habitual place of residence, regardless of their legal status, causes of the movement or whether it was voluntary or involuntary [2]. Worldwide, the unmet health needs of migrants and their lack of access to essential health services are of concern [3]. Consequently, the 2019-2023 draft global action plan on the health of migrants focuses on achieving universal health coverage (UHC) for all people, regardless of citizenship [3]. Some of the key priorities in the draft action plan are the mainstreaming of migrant health into country-level reform agendas, the promotion of migrant-sensitive health policies [3], and the development of health systems that are responsive to their needs [4]. Within the context of UHC, human resources for health (HRH) are central to its achievement [5]. This is because health care providers are the personification of any health system and its responsiveness. Health worker attitudes, behaviour or practices could either advance or constrain the achievement of UHC for vulnerable individuals, such as migrants [6].

In South Africa, there is contestation about the number of migrants, but the 2010 census estimated around 2.2 million immigrants [7]. Legally, there is a constitutional right to health care for all individuals regardless of nationality, but access to health services for migrants is complex, especially for those without formal documentation [6]. This is partly due to the significant challenges faced by government in providing high-quality health care in the public health sector [8]. The proposed National Health Insurance (NHI) system is the country's primary UHC reform, aimed at addressing the

entrenched inequities in its two-tiered health [9]. Although the NHI policy document lacks clarity on health care for migrants and refugees [9], Chapter 2 of the 2019 NHI bill makes provision for coverage of permanent residents, refugees, asylum seekers and conditional cover for "illegal foreigners" [10].

In this paper, we draw on the conceptual framework on social exclusion of the Social Exclusion Knowledge Network (SEKN) to examine the experiences and perspectives of health care providers on migrants utilising public health services in the Gauteng Province of South Africa. The SEKN defines social exclusion as the "dynamic, multidimensional processes driven by unequal power relationships interacting across four main dimensions-economic, political, social and cultural-and at different levels including individual, household, group, community, country and global levels" [11][p36]. In this study, we examined social exclusion at the health system level, specifically the interactions between migrants (a potentially excluded group), and health care providers as the personification of the health system.

Research on migration has focused on the legal instruments for protecting the human rights of migrants and refugees [12], health inequities and unmet health needs of migrants and refugees [13], health policy or system deficiencies [4] and migrant or refugee experiences and perceptions of health services in the host countries [14]. A systematic review on health care provider experiences of care provision to migrants and refugees found major challenges related to diverse cultural beliefs, limited institutional capacity, and the contradiction between health professional ethics and country-specific legislation that limit migrants' right to health care [15]. Another systematic review that explored challenges and facilitators for health professionals providing primary health

care (PHC) for refugees and asylum seekers in high-income countries found that political decisions affect frontline clinical practice, resourcing priorities, health professional roles and healthcare access [16]. The health professionals reported that health care encounters with migrants were influenced by cultural differences, and lack of knowledge of disease conditions in the host country, exacerbated by health system challenges such as a lack of training, insufficient time or professional support to manage complex health problems, referral difficulties, increased costs, and staff shortages [16]. All of these challenges were experienced within a fluid and changing policy environment, and widespread hostility of policymakers [16, 17].

In Africa, a 2018 WHO report underscored the dearth of empirical information on health care to migrants and the ethical responsibilities or professional duties of health care providers [18]. In South Africa, Matlin *et al* [6] have pointed out that despite an enabling legal framework, health care access for migrants is variable in practice and influenced by health system factors, health managers' responsiveness and xenophobic attitudes by health professionals. In a 2011 qualitative study with Zimbabwean migrants in Cape Town and Johannesburg, Crush and Tawodzera coined the term "medical xenophobia", defined as the "negative attitudes and practices of health sector professionals and employees towards migrants and refugees on the job" [19][p.655]. Medical xenophobia included the insistence by managers or health care providers that patients show identity documentation prior to receiving care [19]. It also includes delay or denial of treatment on the basis of nationality, refusal to communicate with patients in a common language (such as English) or to allow the use of translators, and/or verbal abuse and xenophobic statements and insults [19]. A 2017 qualitative study in

Durban, South Africa described the medical xenophobia faced by refugees from the Democratic Republic of Congo (DRC), such as insistence on documentation, insensitive comments and other discriminatory practices from providers [20]. Another, small qualitative study that explored the experiences of eight women refugees and their attempts at utilising reproductive health care services in Durban's public sector also reported incidents of medical xenophobia [21].

However, all of these South African studies were qualitative in design and none of these studies explored the views of health care providers. The aim of this study was to examine the perspectives of health care providers on delivering health services to migrants in public health facilities in the Gauteng Province of South Africa. The paper contributes to an emerging body of literature that examines quality UHC for migrants from the perspective of health care providers.

Material and Methods

Study design and setting

This is a cross-sectional analytical study conducted in public health care facilities in the Gauteng Province of South Africa.

The study setting was all the public health care facilities in Gauteng Province. The province is the most densely populated in South Africa, host to a large number of migrants, and has an estimated total population of 14.7 million [22].

In Gauteng Province, the public health care system consists of four central hospitals, that provide highly specialised quaternary and/or tertiary services, serve as referral

hospitals for lower level facilities, and are attached to university health science faculties that train health professionals [23]. There are also two regional tertiary hospitals that are attached to health science faculties and provide some tertiary and other specialised services, and nine regional hospitals that provide specialised secondary services in internal medicine, general surgery, paediatrics, obstetrics and gynaecology and general surgery. The province has one specialised mother-and child-hospital that functions at the level of a regional hospital, with some tertiary services. The 11 district hospitals in the province provide general, inpatient hospital services, and the six specialised hospitals provide psychiatric services, tuberculosis services, infectious diseases and rehabilitation services [23]. The primary health care (PHC) system consists of a network of 30 community health centres (CHCs) and 290 PHC clinics that provide ambulatory care services. The CHCs tend to have midwife-obstetric units that are open 24 hours per day, seven days per week, while the PHC clinics are day clinics, open from Monday to Friday.

The study population was all health care providers that provide ambulatory care services. This population included medical doctors (both generalists and specialists), professional nurses (with four years of training), enrolled nurses (with two years of training), and nursing auxiliaries or assistants (with one year of training), dentists, occupational therapists, physiotherapists, pharmacists, who render ambulatory care in Gauteng public health care facilities [24].

Sampling of facilities

We used stratified, random sampling to select the public health care facilities from the master list of health care facilities in Gauteng Province (obtained from the Gauteng

Department of Health), which were categorised as follows: central hospital, regional tertiary hospital, regional hospital, district hospital, community health centres, PHC clinics, and a mother and child hospital. We selected two facilities randomly from each stratum, except in the case of the mother and child hospital, where there is only one. Hence we sampled 13 public health care facilities in Gauteng.

Measures

Following an extensive literature review, we designed a self-administered questionnaire that obtained information on the socio-demographic profile of health care providers, and that measured social exclusionary views or practices. We also included an open-ended question to allow for any additional comments on migrants and their health care utilisation, but the qualitative information is excluded from this paper.

The socio-demographic questions elicited information on age, gender, marital status, and whether they have children, category of health care provider, and number of years worked in the health care facility. We measured social exclusionary views or practices among health care providers in two ways: firstly, health care provider experiences of discrimination against, or differential treatment of, migrants (two questions measured by yes or no response); secondly, asking health care providers to rate statements on social exclusionary views or practices (seven questions). The seven statements that measured social exclusionary views or practices of health care providers, focused on examples of medical xenophobia, health professional obligations in relation to migrants, and whether the NHI system should provide cover to migrants. Two of the questions were phrased positively and four were phrased negatively. These were measured using a seven-point Likert scale from strongly disagree (1) to strongly agree (7).

We piloted the questionnaire with five health care providers of different categories at a hospital, clinic and community health centre that were not part of the selected facilities to determine clarity of questions and the time taken to complete the questionnaire. Based on the feedback we received, no changes to the provider SAQ were required.

Data collection

We conducted the study between April and December 2018. At each of the 13 selected public health care facilities, we contacted the health facility manager to plan data collection. For primary health care clinics, we selected three days randomly between Monday and Friday. In the case of community health centres and hospitals, we selected two days randomly between Monday and Friday, and one day randomly on the weekend.

The principal researcher (JW) assisted by another trained fieldworker, recruited eligible health care providers on the randomly selected fieldwork days at each of the selected facilities. The eligibility criteria for participation in the study included a health care provider working in ambulatory care in the hospital outpatient or emergency medical department or in the community health centre and PHC clinics; and the provision of voluntary, informed, written consent.

The research team approached all eligible health care providers on duty on the randomly selected fieldwork days in the health care facilities. We informed potential participants that study participation was voluntary, and that they could withdraw from taking part at any point, without prejudice or negative consequences. Following informed consent, the health care provider completed the self-administered

questionnaire (SAQ) using a tablet, with direct data entry into Research Electronic Data Capture (REDCap), a secure web-based programme hosted at the University of Witwatersrand [25]. The refusal rate was less than 10%.

Statistical analysis

We used STATA® 15 to analyse the data. Frequency tabulations were done to describe the socio-demographic and employment characteristics of the study participants.

The analysis took account of the complex sampling design. All analyses were weighted to reflect the distribution of health care providers, by type of health facility and health worker category, at the provincial level. In the case of the type of facility, we combined: central hospitals, regional tertiary hospitals and specialised mother and child facility into one category, called "tertiary hospitals"; and clinics and community health centres into "primary health care (PHC) facilities". In the case of health care provider, we combined all the enrolled nurses and nursing assistants into one category, called enrolled nurses and nursing assistants.

We used frequency tabulations to show the proportion of health care providers reporting that they witnessed discrimination against, or differential treatment of, migrants. We computed the mean and standard deviations for the 7-point Likert scale items that measured social exclusionary views or practices. Bivariate analysis was done to investigate the relationship between the socio-demographic and employment characteristics of health care providers and each of the responses on social exclusionary views or practices. All the factors found to be statistically significant at a conservative

level of 20% level were included in the multiple regression models. All tests were conducted at 5% significance level.

Ethical considerations

We obtained ethical approval from the Human Research Ethics Committee (Medical) of the University of the Witwatersrand in Johannesburg (Certificate #: M170988). We also obtained permission from the Gauteng Department of Health, district health committees, hospital chief executive officers, and managers of community health centres and PHC clinics. All participants received a detailed study information sheet, and provided written consent, via REDCap (Research Electronic Data Capture) a secure, web-based application for research studies [25]. We complied with the Singapore Declaration of research integrity [26] and adhered to all ethical procedures, including informed consent, voluntary participation, confidentiality and anonymity.

Results

Socio-demographic characteristics of health care providers

A total of 277 health care providers participated in the study (Table 1), the vast majority were women (77.6%), with a mean age of 36.2 (SD 11.4) and a median age of 33 years (range 19-68). The mean age of professional nurses was 45.0 years (SD 11.9), while allied health professionals had a mean age of 28.8 (SD 6.9). Nurses constituted the majority of study participants (51.9%) and the majority were South African (94.8%). A quarter of all study participants worked at central hospitals (25.1%). The mean years worked at any of the selected facilities was 6.8 years (SD 8.4).

Table 1. Demographic and employment characteristics of survey participants

Variable	n	%
**Age** median (range)	33 (19-68)	
**Age by category of health care professional** mean (SD)		
Professional nurses	45.0 (11.9)	
Enrolled nurses and nursing assistants	38.3 (9.6)	
Medical doctors	30.8 (7.1)	
Allied health professionals	28.8 (6.9)	
All participants	36.2 (11.4)	
**Age (years)**		
< 25	42	15.4
25-34	109	40.0
35-44	55	20.1
45-54	35	12.8
55+	32	11.7
**Gender**		
Female	215	77.6
Male	62	22.4
**Place of birth**		

South Africa	254	94.8
Outside South Africa	14	5.2
**Marital status**		
Single	124	44.8
Living together	25	9.0
Married	108	40.0
Divorced/ Widowed	20	7.2
**Category of health care professional**		
Nurses:<br>Enrolled nurses<br>Nursing assistants<br>Professional nurses<br>All categories of nurses	 30<br>33<br>81<br>144	 10.8<br>11.9<br>29.2<br>51.9
Medical doctors	70	25.3
Allied health professionals:<br>Clinical associate<br>Social workers<br>Dieticians/ Dietician assistants<br>Pharmacists/ Pharmacist interns/ Pharmacist assistants<br>Radiographers<br>Rehabilitation therapists (audiologists, speech therapists)	 1<br>2<br>9<br>24<br>6<br>5	 0.4<br>0.7<br>3.2<br>8.7<br>2.2<br>1.8
**Type of health care facility**		
Central hospital	72	25.1
Clinic	27	5.0
Community health centre	24	8.7
District hospital	36	13.0
Regional hospital	65	23.5
Regional Tertiary hospital	51	18.4
Specialised Mother & Child hospital	15	5.4
**Median years (range) worked in facility**<br>**Mean years (SD) worked in facility**	3 (0.08-39)<br>6.8 (8.4)	

**Number of years worked in facility**		
< 2 years	82	29.6
2-4 years	81	29.2
5-9 years	57	20.6
10-14 years	20	7.2
15+ years	37	13.4

Health care provider reported discrimination or differential treatment

The majority of health care providers surveyed (79.0%) reported that they did not witness any discrimination nor differential treatment (77.3%) of migrants in their work settings (Fig.1). Although more medical doctors reported that they witnessed discrimination (31.4%) or differential treatment (31.4%) compared to other categories of health professional (Fig. 1). When compared using a chi-square test, there was no association between health care providers reporting of witnessed discrimination (x^2 = 4.97; p = 0.11) and differential treatment (x^2 = 5.08; p = 0.39).

INSERT FIG.1 HERE

Social exclusionary views or practices

Table 2 shows health care providers' mean scores of social exclusionary views or practices by total score, socio-demographic and employment characteristics. Items (or statements) are arranged in the table from positively worded statements on the left to negatively worded statements on the right. More exclusionary scores are indicated by disagreement with 3 positive statements (scores 1-3) and agreement with 4 negative statements (scores 4-7).

Positively-worded statements

Providers obtained an overall mean score of 4.4 for the item on being sensitive to the health care needs of migrants and refugees. Providers reporting more exclusionary views for this item, included: the age category of 45 – 54 years (M 3.8; SD 1.8); enrolled nurses and nursing assistants (M 3.7; SD 1.6) and those working in a health care facility for a period of 5 – 9 years (M 3.8; SD 2.0). Conversely, providers born

outside of South Africa (M 6.5; SD 0.8); medical doctors (M 5.5; SD 2.1) and allied health professionals (M 5.1; SD 3.1) showed less exclusionary views.

The highest overall mean score was obtained for the item on providing the same quality of care to migrants and refugee as to South Africans was 6.1 (SD 1.5). Similar mean scores were reported from participants in the age categories 35 – 44 years (M 5.8; SD 1.6) and 45 – 54 years (M 5.9; SD 1.5); those who were single (M 5.8; SD 1.9); and enrolled nurses/ nursing assistants (M 5.8; SD 1.3). Providers working in a health care facility for periods between 10 and 14 years (M 5.7; SD 1.1) and periods of more than 15 years (M 5.9; SD 1.5) also showed less exclusionary views.

Providers obtained an overall mean score of 3.4 (SD 2.0) for the item on NHI coverage for migrants and refugees, indicating more exclusionary views. Similarly, providers aged 45-54 years (M 2.8; SD 1.7), those who are allied health professionals (M 2.7; SD 2.6), and those working in the facility for 10-14 years (M 2.8; SD 1.8) also reported more exclusionary views. The highest mean score – indicating less exclusionary views - was obtained for providers born outside of South Africa (M 5.2; SD 2.1).

Negatively-worded statements

Overall, providers showed less exclusionary views for the item on discriminating against migrant and refugee patients with a mean score of 1.7 (SD 1.1) (Table 2). Mean scores for this item did not vary by much from the overall mean score, with the highest obtained for providers working in a health care facility between 10-14 years (M 2.3; SD 1.0), still indicative of less exclusionary views.

Providers showed less exclusionary scores for the item on delaying health care to patients because of their migration status, with an overall mean score of 1.7 (SD 1.2) (Table 2). Similarly, other mean scores for this item also showed less exclusionary views, with the highest mean score being 2.2 (SD 1.5), obtained from providers in the age category of 55 years and older.

Overall, providers obtained a mean score of 3.4 (SD 2.1) for the item that migrants and refugees should return to their home country for health care. Slightly lower mean scores were obtained for: male participants (M 2.6; SD 1.9); those younger than 25 years old (M 2.7; SD 2.5), those born outside of South Africa (M 1.4; SD 1.6); medical doctors (M 2.7; SD 2.3) and allied health professionals (M 2.5; SD 2.8).

Providers obtained an overall mean score of 4.0 (SD 2.2) for the item on migrants and refugees only coming to South Africa for health care services. Providers born outside South Africa (M 2.6; SD 2.2); and allied health professional (M 4.2; SD 3.0) reported less exclusionary views for this item.

Table 2: Table 2: Providers' mean scores (standard deviations) of social exclusionary views or practices by socio-demographic and employment characteristics

Variable		I am sensitive to the health care needs of migrants and refugees. Mean (SD)	I provide the same quality of care to migrants and refugees as I do to South Africans. Mean (SD)	I believe migrants and refugees should be covered under the NHI. Mean (SD)	I discriminate against migrant and refugee patients. Mean (SD)	I have delayed health care to patients because of their migrant or refugee status. Mean (SD)	I believe migrant and refugee patients should go back to their home country for health care. Mean (SD)	I believe that migrant and refugee patients only come to South Africa for health care services. Mean (SD)
**Total**		4.4 (2.1)	6.1 (1.5)	3.4 (2.0)	1.7 (1.1)	1.7 (1.2)	3.4 (2.1)	4.0 (2.2)
**Gender**	Male	4.5 (2.1)	6.0 (1.8)	4.0 (2.2)	1.5 (1.0)	1.5 (1.2)	2.6 (1.9)	3.1 (2.2)
	Female	4.4 (2.1)	6.1 (1.4)	3.3 (2.0)	1.7 (1.1)	1.7 (1.2)	3.6 (2.1)	4.1 (2.1)
**Age Group**	< 25 years	4.9 (2.7)	6.7 (0.7)	4.0 (2.7)	1.1 (0.4)	1.3 (1.0)	2.7 (2.5)	3.7 (2.3)
	25-34 years	4.5 (2.0)	6.1 (1.6)	3.7 (2.1)	1.7 (1.3)	1.6 (1.1)	3.3 (2.1)	3.5 (2.4)
	35-44 years	4.2 (2.1)	5.8 (1.6)	3.4 (2.0)	1.7 (0.7)	1.5 (0.6)	3.2 (2.1)	4.1 (2.0)
	45-54 years	3.8 (1.8)	5.9 (1.5)	2.8 (1.7)	2.0 (1.0)	2.0 (1.2)	3.7 (2.0)	4.5 (1.8)
	55+ years	4.7 (1.8)	6.2 (1.1)	3.4 (1.7)	1.7 (0.8)	2.2 (1.5)	4.0 (1.7)	4.0 (2.0)
**Origin**	Born in South Africa	4.3 (2.0)	6.1 (1.5)	3.3 (2.0)	1.7 (1.1)	1.7 (1.2)	3.5 (2.1)	4.0 (2.2)
	Born outside South Africa	6.5 (0.8)	6.0 (2.2)	5.2 (2.1)	1.3 (0.5)	1.2 (0.5)	1.4 (1.6)	2.6 (2.2)
**Marital Status**	Single	4.2 (2.2)	5.8 (1.9)	3.2 (1.9)	1.7 (1.0)	1.7 (1.3)	3.5 (2.2)	4.2 (2.3)
	Living together	4.5 (1.8)	6.5 (0.9)	4.0 (2.3)	1.6 (1.1)	1.5 (0.6)	3.3 (2.0)	3.3 (1.9)
	Married	4.5 (2.2)	6.3 (1.2)	3.8 (2.2)	1.6 (0.9)	1.8 (1.3)	3.2 (2.2)	3.8 (2.2)
	Divorced/Widowed	4.2 (1.7)	6.0 (1.2)	3.4 (1.7)	2.0 (1.0)	1.8 (1.0)	3.7 (1.8)	2.6 (1.6)
**HCP Category**	Professional nurse	4.5 (1.9)	6.1 (1.4)	4.3 (2.0)	1.6 (0.8)	1.7 (1.1)	3.8 (2.0)	3.2 (1.8)
	Enrolled nurse/ Nursing assistant	3.7 (1.6)	5.8 (1.3)	4.0 (1.7)	2.0 (1.0)	2.0 (1.0)	3.4 (1.6)	3.4 (1.6)
	Medical doctor	5.5 (2.1)	6.4 (1.5)	3.4 (2.4)	1.3 (0.9)	1.3 (0.9)	2.7 (2.3)	3.8 (2.6)
	Allied health professional	5.1 (3.1)	6.6 (1.4)	2.7 (2.6)	1.3 (1.0)	1.3 (1.2)	2.5 (2.8)	4.2 (3.0)
**Type of facility**	Tertiary hospital	4.2 (2.1)	6.0 (1.6)	4.1 (2.3)	1.8 (1.1)	2.0 (1.5)	3.6 (2.2)	3.2 (2.1)
	Regional hospital	4.5 (2.2)	6.0 (1.3)	3.8 (2.1)	1.8 (1.2)	1.6 (0.8)	3.0 (2.0)	3.5 (2.0)
	District hospital	4.2 (1.8)	6.4 (0.5)	3.4 (2.0)	1.4 (0.6)	1.5 (0.9)	3.2 (1.9)	3.9 (2.1)
	PHC facilities	5.0 (2.1)	6.0 (2.0)	3.8 (2.1)	1.3 (0.6)	1.4 (1.0)	3.6 (2.2)	3.8 (1.8)
**Years working at facility**	< 2 years	5.1 (2.3)	6.0 (2.3)	3.8 (2.6)	1.4 (1.2)	1.4 (1.1)	3.1 (2.7)	3.5 (2.5)
	2-4 years	4.6 (2.0)	6.3 (1.1)	3.5 (2.0)	1.6 (1.2)	1.5 (1.0)	3.0 (2.0)	3.5 (2.2)
	5-9 years	3.8 (2.0)	6.1 (1.3)	3.7 (2.0)	1.7 (0.8)	1.8 (1.1)	3.4 (1.9)	4.4 (2.0)
	10-14 years	4.4 (1.6)	5.7 (1.1)	2.8 (1.8)	2.3 (1.0)	2.0 (1.0)	4.6 (1.9)	4.5 (1.9)
	15+ years	4.4 (1.6)	5.9 (1.5)	3.0 (1.5)	1.7 (0.9)	2.0 (1.3)	3.7 (1.8)	4.0 (1.8)

Predictors of social exclusionary views or practices among health care providers

Table 4 shows the results of the multiple regression analyses on the predictors of social exclusionary views or practices among health care providers. A negative co-efficient on the positively worded statements (first three in table) and a positive co-efficient on the negatively worded statements (last four in table) indicates more exclusionary views (Table 4).

Participants born outside of South Africa had a significantly higher score ($p < 0.05$) than those born in South Africa on being sensitive to the health care needs of migrant and refugee patients, indicative of less exclusionary views. Enrolled nurses and nursing assistants had a significantly lower score ($p<0.001$) on being sensitive to the health care needs of migrant and refugee patients, indicating more exclusionary views.

Participants aged 35-44 years and enrolled nurses and nursing assistants held more exclusionary views on providing the same quality of care to migrant and refugee patients ($p<0.05$). With regard to the inclusion of migrants under the NHI, single participants had significantly lower mean scores ($p<0.05$), suggesting relatively more exclusionary views with regard to NHI coverage. Gender, age, category of health care professional, and years worked in health care facility were no longer significant contributors in the regression model.

Providers aged 25-34 years, 55 years and older, and enrolled nurses and nursing assistants were significantly more likely to indicate agreement with having discriminated against migrant patients ($p<0.05$). In contrast, providers working in district hospitals, in

PHC facilities; and providers working in the health care facility for a period of between 10-14 years agreed less with having discriminated against migrant patients ($p<0.05$).

Category of health care professional and type of health care facility were significant predictors of participants' views on delaying care because of migration status. In particular, enrolled nurses and nursing assistants had a significantly higher score indicating agreement with delaying care because of migration status ($p<0.05$). On the other hand, participants who disagreed with this view on delaying care included providers working in regional hospitals ($p<0.001$), district hospitals ($p<0.05$), PHC facilities ($p<0.05$).

In terms of the view that migrants and refugees should return to their home country for health services, gender, category of health care professional, and place of birth were predictors. Female participants, professional nurses, enrolled nurses and nursing assistants had significantly higher scores than any other category of health care professional ($p<0.05$). Providers born outside of South Africa had significantly lower scores than those born in South Africa ($p<0.001$), indicating less exclusionary views.

Female providers had a significantly higher score than male providers on the view that migrants only come to South Africa for health care services ($p<0.05$). Similarly, single participants also held more exclusionary views for this item than others in the marital status category ($p<0.05$). Professional nurses, ($p<0.001$), enrolled nurses and nursing assistants ($p<0.05$), and medical doctors ($p<0.05$) had higher scores, indicating agreement with this view on migrants only coming to South Africa for health care.

Table 3: Predictors of social exclusionary views or practices among health care providers

Variable		I am sensitive to the health care needs of migrants and refugees.		I provide the same quality of care to migrants and refugees as I do to South Africans.		I believe migrants and refugees should be covered under the NHI.		I discriminate against migrant and refugee patients.		I have delayed health care to patients because of their migrant or refugee status.		I believe migrant and refugee patients should go back to their home country for health care.		I believe that migrant and refugee patients only come to South Africa for health care or services.	
		β	p-value	β	p-value	β	p-value	β	p-value	β	p-value	β	p-value	β	p-value
**Gender**	Reference: Male														
	Female	-	-	-	-	-0.65	0.18	0.15	0.37	0.09	0.73	0.95	0.005*	0.87	0.033*
**Age Group**	Reference: < 25 years														
	25-34 years	0.27	0.49	-0.50	0.08	-0.32	0.28	0.46	0.016*	0.04	0.83	0.40	0.36	-0.46	0.36
	35-44 years	0.04	0.94	-0.77	0.040*	-0.71	0.12	0.18	0.34	-0.10	0.75	-0.02	0.96	-0.03	0.97
	45-54 years	0.23	0.69	-0.70	0.16	-1.19	0.07	0.48	0.05	0.29	0.50	0.10	0.86	0.40	0.51
	55+ years	1.36	0.16	-0.35	0.38	-0.45	0.51	0.59	0.004*	0.78	0.11	0.55	0.50	0.02	0.98
**Marital Status**	Reference: Married														
	Single	-	-	0.22	0.56	-0.82	0.010*	-	-	0.00	0.98	-	-	0.69	0.045*
	Living together	-	-	-0.60	0.17	0.01	0.98	-	-	-0.13	0.65	-	-	-0.32	0.45
	Divorced/Widowed	-	-	-0.33	0.46	-0.74	0.23	-	-	-0.25	0.57	-	-	-0.78	0.30
**Born in/ out South Africa**	Reference: Born in SA														
	Born outside SA	1.58	0.001*	-	-	-	-	0.01	0.97	-0.02	0.92	-1.36	p<0.001	-1.17	0.026*
**HCP Category**	Reference: Allied health professional														
	Professional nurse	-0.64	0.13	-0.48	0.08	-0.77	0.15	0.00	0.98	0.21	0.32	1.23	0.029*	1.91	0.001*
	Enrolled nurses & nursing assistants	-1.32	p< 0.001	-0.73	0.010*	-0.64	0.18	0.54	0.012*	0.49	0.030*	1.13	0.009*	1.42	0.033*
	Medical doctor	-0.13	0.67	-0.19	0.44	-0.56	0.23	-0.08	0.71	0.06	0.78	0.69	0.22	1.65	p<0.001
**Type of facility**	Reference: Tertiary hospital														
	Regional hospital	-	-	0.10	0.72	-	-	0.01	0.98	-0.40	p<0.001	-	-	-	-
	District hospital	-	-	0.45	0.06	-	-	-0.46	0.015*	-0.37	0.010*	-	-	-	-
	PHC facility	-	-	-0.00	0.99	-	-	-0.37	0.042*	-0.52	0.001*	-	-	-	-
**Years working at facility**	Reference: < 2 years														
	2-4 years	-0.06	0.81	-	-	-0.21	0.61	-0.08	0.66	-0.07	0.73	-0.55	0.25	0.01	0.98
	5-9 years	-0.46	0.27	-	-	0.41	0.48	-0.10	0.58	0.13	0.74	-0.26	0.65	0.63	0.27
	10-14 years	-0.01	0.98	-	-	-0.31	0.64	0.58	0.027*	0.31	0.40	0.72	0.20	0.45	0.52
	15 or more years	-1.16	0.21	-	-	0.01	0.98	-0.23	0.59	-0.18	0.74	-0.41	0.34	-0.26	0.38
**Constant**		5.13	p<0.001	7.26	0.000**	5.55	p<0.001	3.73	0.001	1.43	0.018	1.73	0.001	1.54	0.022

Only predictor variables statistically significant at 20% in the bivariate analysis were included in the multiple regression models. *p<0.05

Discussion

This is one of the first surveys in South Africa, and indeed in Africa, that examined the perspectives of health care providers on public health care services to migrants. Most of the study participants were female (77.6%) and nurses (51.9%). This is not surprising as the majority of health care providers in South Africa are nurses, and women [27]. Our study showed that predictors of providers' more exclusionary or less exclusionary views included female gender, single status, 25 years and older, category of health care provider, type of facility and years worked in a health care facility.

In our study, providers obtained a score of 3.4 for the item on sensitivity to the health needs of migrants; and a score of 6.1 for providing the same quality of care. As with a previous study, we showed that providers born outside South Africa reported greater sensitivity to the needs of migrants [28]. This finding makes sense because these providers are themselves migrants. The Canadian study showed that those providers born outside Canada expressed greater cultural sensitivity and were more comfortable with immigrant patients compared to their counterparts from Canada [28]. In contrast, enrolled nurses and nursing assistants in our study had lower scores for sensitivity to the needs of migrants, suggesting more exclusionary views. Possible explanations for these exclusionary views could be related to insufficient or lack of training on culturally-responsive health care, as enrolled nurses undergo two years of training and nursing assistants undergo only one year of training. Other studies in Canada and Australia, albeit with physicians, found that cultural barriers mitigated against the provision of migrant-sensitive health care services [29-31]. Several studies in Canada and Australia demonstrated the benefits to migrant patient outcomes when health care providers

received culture-sensitivity training [32], as the training enhanced their expressed sensitivity [33, 34], empathy [35] and cultural humility [36]. Although the context of these studies cited are different from that of South Africa, there would be value in ethics and culture-sensitivity training for health care providers in the South African public service, focusing on enrolled nurses and nursing assistants.

The overall mean score for the item on discrimination against migrants was 1.7. This means that health care providers in our study indicated disagreement with this statement. Similarly, the mean score for delayed care to migrants - a form of discrimination - was 1.7. These responses are encouraging. In the regression analysis, providers aged 25-34, older than 55, enrolled nurses and nursing assistants, and working in a health care facility for between 10 and 14 years reported greater agreement with this discriminatory statement, and thus more exclusionary views. Interestingly, working in a district hospital and PHC facility was a predictor of less exclusionary views. Of additional concern is that one in five health care providers (21.0%) reported that they had witnessed discrimination, and 22.6% reported that they had witnessed differential treatment of migrants in their work settings. These reported discrimination and differential treatment are an example of the attitudes and behaviours associated with medical xenophobia in the South African public health system [37]. A South African study among medical students found that 10% reported that they had witnessed discriminatory behaviour of providers against patients on the basis of race, ethnicity or age [38]. Studies in Europe [39, 40] that examined health care providers' experiences of discrimination found both a reluctance to talk about discrimination, and evidence of discriminatory attitudes towards migrant patients. However, another study

in Greece found a mixed picture of the interaction between providers and migrants, with some providers prioritizing the health care of citizens over migrants, while others provided unrestricted health care access to undocumented migrants despite restrictive laws [41].

South Africa's Constitution outlaws discrimination [42] because of the country's apartheid history, where there were gross violations of the rights of black people [43]. Health care providers are required to uphold professional and ethical standards of care [44, 45]. The various health professional Oaths emphasis service to humanity, practising with conscience and with dignity, pursuing justice and advocating on behalf of vulnerable and disadvantaged patients [46, 47]. Although the majority of health care providers in our study appear to meet their professional obligations, it is unacceptable that a minority of providers expressed social exclusionary views. A combination of strategies is needed to ensure that migrant-sensitive health services are provided, and that all patients in the Gauteng public health service are treated with respect and dignity, regardless of nationality. These strategies include advocacy training and campaigns that emphasise the rights and responsibilities of providers, engagement of civil society organisations, and clear communication about the complaints mechanisms, including the toll-free number of the Health Ombud [48]. There should also be adverse consequences for those health care providers that continue discrimination against migrants, and they should be reported to the relevant health professions council for possible disciplinary action.

The mean score for the item that migrants should return to their home country for health care was 3.4, and that migrants only come to South Africa for health care was

4.0. Female providers, professional nurses and enrolled nurses and nursing assistants had significantly higher agreement than the other categories, suggesting more social exclusionary views. These views could explain the mean score of 3.4 for NHI coverage for migrants, suggesting that participants did not agree with the inclusion of migrants and refugees in the proposed NHI. In the regression, single status was the only predictor of a more exclusionary view. It is unclear why single participants had these views and highlight this finding as an unusual finding in our study. Despite this anomaly, it is still of concern that more health care providers hold this view, given that they have a critical role to play in the achievement of UHC [49]. Scholars have suggested that health care providers work in constrained conditions, exacerbated by migrant-unfriendly regulatory frameworks, policies and political rhetoric [50]. This context might explain the social exclusionary views of some of these health workers surveyed views. Moreover, the understanding that migrants only come to South Africa for health care may not necessarily be indicative of exclusionary views. In other words, it may reflect the reality of migrants who because of virtual collapse of health care in home countries, such as the case in Zimbabwe, will make decisions about survival and that is to seek health care in South Africa.

The study is limited by its cross-sectional nature, and the self-reported information obtained from health care providers. However, the self-administered questionnaire using tablets, allowed providers to express their views in a confidential manner. The study was only conducted in Gauteng Province, and the findings might not apply to other provinces. However, there are numerous study strengths. This is one of the first surveys that examined the views of health care providers on migrants utilising public

health facilities, rather than the systemic barriers or challenges in the interactions between migrants and providers [15, 16, 51]. The random sampling of facilities and fieldwork days gives robustness to our findings. The questionnaire could be adapted by other researchers in similar settings, and complemented with qualitative research to add depth to the quantitative findings. Future research could include a larger sample of public health facilities in Gauteng, as well as comparisons with other provinces.

As South Africa moves towards the implementation of the NHI, discrimination and other social exclusionary views or practices of health care providers will undermine progress. The United Nations has called on Member States to put an end to discrimination of migrants in health care settings, through a joint statement outlining three key priority areas of action: supporting the rights of both patients and providers; tackling discrimination through an evidence base and appropriate legal frameworks that ensure accountability; and lastly, collaboration between governments, civil society and communities to address the determinants of discrimination [52]. Health care providers are the foundation of quality UHC. Our study findings suggest the complexity of the interaction between providers and migrant patients in Gauteng rather than a straightforward or "single" narrative. Given its economic importance, Gauteng should take the lead in implementing the UN recommendations, and to develop more inclusive health policies to the benefit of all patients, in support of the achievement of UHC.

Conclusion

Health care providers, specifically medical doctors and nurses, are at the front-line of health care delivery and are thus integral to the provision of migrant-sensitive health care. Given this criticality, providers' perspectives on social exclusionary views or

practices are important in shaping inclusive health policies. Using a lens of social exclusion, we have generated new knowledge on health care provider-migrant interactions in Gauteng Province. This study has shown that health care providers, in most instances enrolled nurses and nursing assistants, have significantly more exclusionary views of migrants within the context of health care services. Social exclusionary views or practices must be addressed at all levels through a multi-pronged approach, that includes training in culture-sensitivity, ethics and human rights; promoting health care providers as advocates for migrant patients and their rights; and recognising the importance of an enabling health system.

Acknowledgements

We acknowledge Dr Bridget Ikalafeng for facilitating permission at Provincial level to conduct the study. We also thank the CEOs and facility managers for permission to conduct the study at facilities and the ease of access their support provided. Professor Jonathan Levin and Dr Innocent Maposa are acknowledged for their statistical inputs. We acknowledge the 5 fieldworkers who assisted with data collection. A special acknowledgement is given to the providers who participated in the study.

Author Contributions

Author Contributions Conceptualization: Janine Anthea White, Laetitia Charmaine Rispel.

Data curation: Janine Anthea White.

Formal analysis: Janine Anthea White, Duane Blaauw, Laetitia Charmaine Rispel.

Funding acquisition: Janine Anthea White.

Investigation: Janine Anthea White.

Methodology: Janine Anthea White, Laetitia Charmaine Rispel.

Project administration: Janine Anthea White.

Supervision: Laetitia Charmaine Rispel.

Validation: Janine Anthea White.

Visualization: Janine Anthea White.

Writing – original draft: Janine Anthea White.

Writing – review & editing: Janine Anthea White, Duane Blaauw, Laetitia Charmaine Rispel.

References

1. Internal Displacement Monitoring Centre. IDMC Grid 2016 – Global Report on Internal Displacement: IDMC; 2016 [22 May 2019]. Available from: http://www.internal-displacement.org/globalreport2016/.
2. United Nations High Commissioner for Refugees. Global Appeal 2018/2019. Geneva: 2019.
3. World Health Organization. Promoting the health of refugees and migrants: Draft global action plan, 2019–2023. Report by the Director-General. Seventy-second World Health Assembly, A72/25, Provisional agenda item 12.4, 25 April 2019. Geneva: World Health Organization, 2019.
4. World Health Organization. Beyond the barriers Framing evidence on health system strengthening to improve the health of migrants experiencing poverty and social exclusion. Geneva: 2017 9789241513364.
5. World Health Organization. Global strategy on human resources for health: Workforce 2030. Geneva: World Health Organization, 2016.
6. Matlin SA, Depoux A, Schütte S, Flahault A, Saso L. Migrants' and refugees' health: towards an agenda of solutions. Public Health Reviews. 2018;39:27:https://doi.org/10.1186/s40985-018-0104-9.
7. Statistics South Africa. Census 2011. Statistical release (Revised). Pretoria: 2011.
8. South African Lancet National Commission. Confronting the right to ethical and accountable quality health care in South Africa: A Consensus Report. Pretoria National Department of Health 2019.
9. NDoH. National Health Insurance Policy: Towards universal health coverage. Pretoria, Republic of South Africa: National Department of Health (NDoH), 2017.
10. Republic of South Africa. National Health Insurance Bill, (2019).
11. Popay J, Escorel S, Hernandez M, Johnston H, Mathieson J, Rispel L, on behalf of the WHO Social Exclusion Knowledge Network. Understanding and Tackling Social Exclusion: Final Report of the Social Exclusion Knowledge Network of the Commission on Social Determinants of Health. Geneva: World Health Organization, 2008.
12. Lori JR, Boyle JS. Forced migration: Health and human rights issues among refugee populations. Nursing Outlook. 2015;63(1):68-76. doi: 10.1016/J.OUTLOOK.2014.10.008.
13. World Health Organization. How health systems can address health inequities linked to migration and ethnicity. Copenhagen: 2010.
14. Human Rights Watch. No Healing Here: Violence, Discrimination and Barriers to Health for Migrants in South Africa [Internet]. New York: Human Rights Watch, 2009.
15. Suphanchaimat R, Kantamaturapoj K, Putthasri W, Prakongsai P. Challenges in the provision of healthcare services for migrants: A systematic review through providers' lens. BMC Health Services Research. 2015;15:1-14. doi: 10.1186/s12913-015-1065-z.
16. Robertshaw L, Dhesi S, Jones LL. Challenges and facilitators for health professionals providing primary healthcare for refugees and asylum seekers in high-income countries: a systematic review and thematic synthesis of qualitative research. BMJ Open. 2017;7(8):e015981. doi: 10.1136/bmjopen-2017-015981.

17. Smith AC, LeVoy M. Cities of rights: Ensuring health care for undocumented residents. Brussels: The Platform for International Cooperation on Undocumented Migrants (PICUM), 2017.
18. WHO AFRO. Health of refugees and migrants:Practices in addressing the health needs of refugees and migrants. Brazzaville: World Health Organization Regional Office for Africa; 2018.
19. Crush J, Tawodzera G. Medical Xenophobia: Zimbabwean access to health services in South Africa. Cape Town: Southern African Migration Programme (SAMP) & Open Society Initiative for Southern Africa (OSISA), 2011.
20. Zihindula G, Meyer-Weitz A, Akintola O. Lived experiences of Democratic Republic of Congo refugees facing medical xenophobia in Durban, South Africa. Journal of Asian and African Studies. 2017;52(4):458-70.
21. Munyaneza Y, Mhlongo EM. Medical Xenophobia: The Voices of Women Refugees in Durban, Kwazulu-Natal, South Africa. BioRxiv. 2019:603753.
22. Statistics South Africa. Mid-year population estimates 2018. Pretoria: Statistics South Africa, 2018.
23. Republic of South Africa. National Health Act, Stat. 35101 (2003).
24. Hassim A, Heywood M, Berger J. Health & democracy : a guide to human rights, health law and policy in post-apartheid South Africa. Johannesburg: SiberInk; 2007. 520 p.
25. Harris PA, Taylor R, Thielke R, Payne P, Gonzalez N, Conde JG. Research electronic data capture (REDCap) - A metadata-driven methodology and workflow process for providing translational research informatics support J Biomed Inform. 2009;42(2):377-81.
26. Singapore statement on research integrity. World Conference on Research Integrity; 2010; Singapore.
27. Department of Health. Strategic Plan for Nursing Education, Training and Practice 2012/13–2016/17. Pretoria: Department of Health (DOH), 2013.
28. Papic O, Malak Z, Rosenberg E. Survey of family physicians' perspectives on management of immigrant patients: Attitudes, barriers, strategies, and training needs. Patient Education and Counseling. 2012;86(2):205-9. doi: 10.1016/j.pec.2011.05.015.
29. Scheppers E, Van Dongen E, Dekker J, Geertzen J, Dekker J. Potential barriers to the use of health services among ethnic minorities: a review. Family practice. 2006;23(3):325-48.
30. Uiters E, Devillé W, Foets M, Spreeuwenberg P, Groenewegen PP. Differences between immigrant and non-immigrant groups in the use of primary medical care; a systematic review. BMC health services research. 2009;9(1):76.
31. Farley R, Askew D, Kay M. Caring for refugees in general practice: perspectives from the coalface. Australian Journal of Primary Health. 2014;20(1):85-91.
32. Majumdar B, Browne G, Roberts J, Carpio B. Effects of Cultural Sensitivity Training on Health Care Provider Attitudes and Patient Outcomes. Journal of Nursing Scholarship. 2004;36(2):161-6. doi: 10.1111/j.1547-5069.2004.04029.x.
33. Lawrence J, Kearns R. Exploring the 'fit'between people and providers: refugee health needs and health care services in Mt Roskill, Auckland, New Zealand. Health & social care in the community. 2005;13(5):451-61.

34. Suurmond J, Seeleman C, Rupp I, Goosen S, Stronks K. Cultural competence among nurse practitioners working with asylum seekers. Nurse education today. 2010;30(8):821-6.
35. Burchill J, Pevalin DJ. Demonstrating cultural competence within health-visiting practice: working with refugee and asylum-seeking families. Diversity and Equality in Health and Care. 2014;11(2):151-9.
36. Tobin CL, Murphy-Lawless J. Irish midwives' experiences of providing maternity care to non-Irish women seeking asylum. International journal of women's health. 2014;6:159.
37. Crush J, Tawodzera G. Medical Xenophobia and Zimbabwean Migrant Access to Public Health Services in South Africa. Journal of Ethnic and Migration Studies. 2014;40(4):655-70. doi: 10.1080/1369183X.2013.830504.
38. Vivian LMH, Naidu CS, Keikelame MJ, Irlam J. Medical students' experiences of professional lapses and patient rights abuses in a South African health sciences faculty. Academic Medicine. 2011;86(10):1282-7. doi: 10.1097/ACM.0b013e31822be4b8.
39. European Union Agency for Fundamental Rights. Inequalities and multiple discrimination in access to and quality of healthcare Luxembourg: 2013.
40. Papadakaki M, Lionis C, Saridaki A, Dowrick C, de Brún T, O'Reilly-de Brún M, et al. Exploring barriers to primary care for migrants in Greece in times of austerity: Perspectives of service providers. European Journal of General Practice. 2017;23(1):129-35. doi: 10.1080/13814788.2017.1307336.
41. Teunissen E, Tsaparas A, Saridaki A, Trigoni M, van Weel-Baumgarten E, Van Weel C, et al. Reporting mental health problems of undocumented migrants in Greece: A qualitative exploration. European Journal of General Practice. 2016;22(2):119-25.
42. Republic of South Africa. Constitution of the Republic of South Africa 1996, Act 108 of 1996. URL: http://www.info.gov.za: 1996.
43. Coovadia H, Jewkes R, Barron P, Sanders D, McIntyre D. The health and health system of South Africa: historical roots of current public health challenges. Lancet. 2009;374:817-34. doi: http://thelancet.com.
44. Dhai A. The Life Esidimeni tragedy: Moral pathology and an ethical crisis. South African Medical Journal. 2018;108(5):382-5.
45. Lown BA. Compassion Is a Necessity and an Individual and Collective Responsibility. Comment on "Why and How Is Compassion Necessary to Provide Good Quality Healthcare?". International Journal of Health Policy and Management. 2015;4(9):613-4. doi: 10.15171/ijhpm.2015.110. PubMed PMID: 26340491.
46. World Medical Association. Declaration of Geneva May 2006 [cited 2019 25 November]. Available from: https://www.wma.net/policies-post/wma-declaration-of-geneva/.
47. SANC. Nurses' Pledge n.d. [cited 2019 25 November]. Available from: https://www.sanc.co.za/aboutpledge.htm.
48. Office of the Health Ombud. Official website of the Office of the Health Ombud [cited 2019 25 November]. Available from: http://healthombud.org.za/.
49. World Health Organization. Draft thirteenth general programme of work, 2019–2023. Report by the Director-General. World Health Assembly Seventy-first World Health Assembly, 71/4, Provisional agenda item 11.1. Geneva: World Health Organization, 2018.

50. Vearey J, Modisenyane M, Hunter-Adams J. Towards a migration-aware health system in South Africa: a strategic opportunity to address health inequity. In: Padarath A, Barron P, editors. South African Health Review 2017. Durban: Health Systems Trust; 2017.
51. Ahmed S, Shommu NS, Rumana N, Barron GRS, Wicklum S, Turin TC. Barriers to Access of Primary Healthcare by Immigrant Populations in Canada: A Literature Review. Journal of Immigrant and Minority Health. 2016;18(6):1522-40. doi: 10.1007/s10903-015-0276-z.
52. United Nations. Joint United Nations statement on ending discrimination in health care settings. 2017.

Figure Click here to download Figure Fig 1.tiff

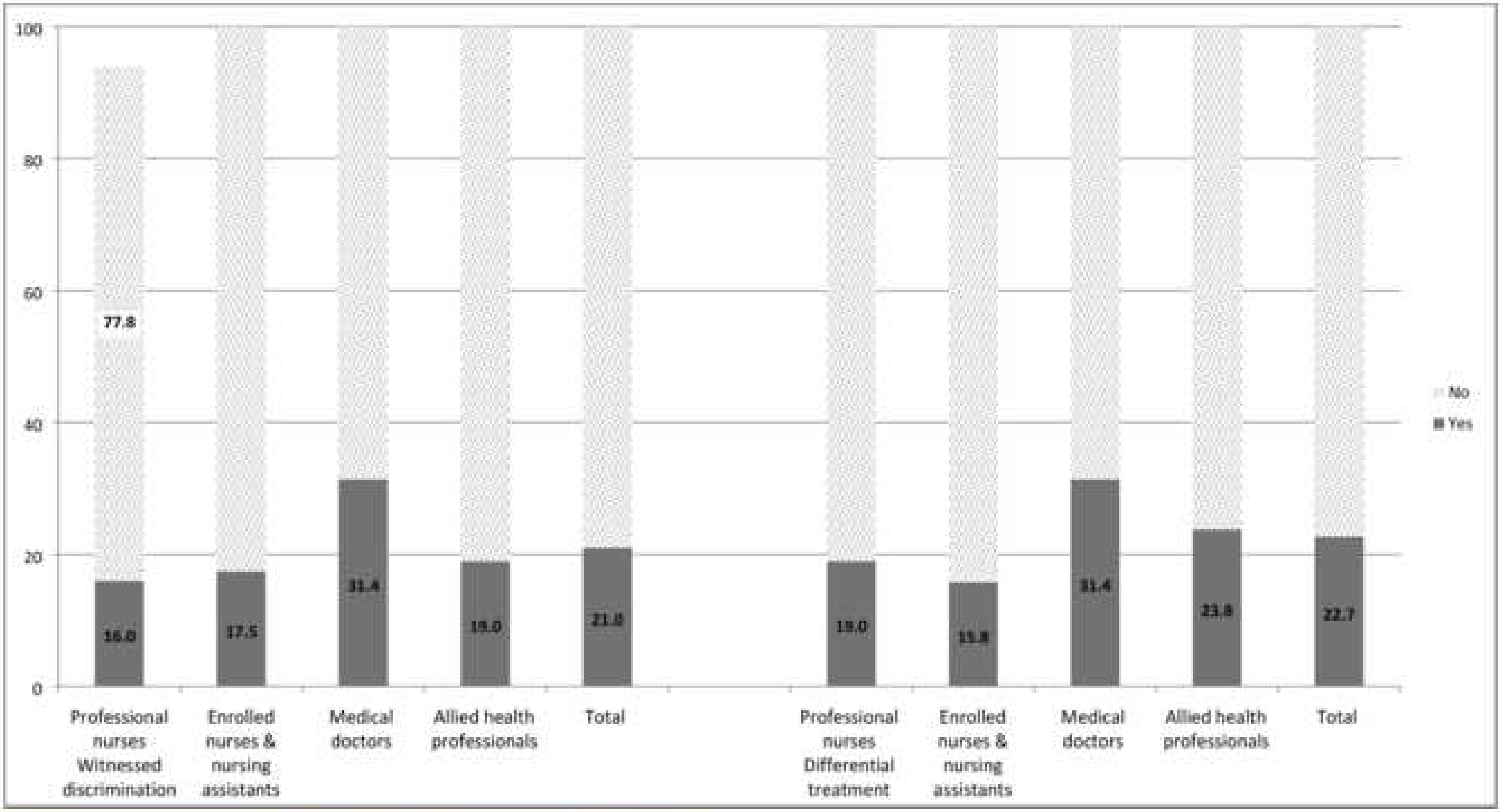

Figure 1: Reported discrimination or differential treatment witnessed by health care providers
