# Supplementary material for: Social exclusion and the perspectives of health care providers on migrants in Gauteng public health facilities, South Africa
Source: PLoS One. 2020 Dec 28;15(12):e0244080. doi: 10.1371/journal.pone.0244080 (PMC7769451; doi:10.1371/journal.pone.0244080)
Supplement: Attachment — Submitted filename: Response to PLOS reviewers 18 Nov 2020_.docx [file pone.0244080.s003.docx]

Faculty of Health Sciences, 27 St Andrew’s Road, Parktown 2193; Johannesburg; South Africa T +27 (0) 11 717 2259

18 November 2020

Editor-in-chief

PLOS ONE

**RE: RESPONSE TO REVIEWERS-PLOS ONE DECISION: Revision required [PONE-D-19-34029R1] - [EMID:f3691ac41a5b1b36]**

We thank you and the reviewers’ for the comments on the manuscript entitled: *Social exclusion and the perspectives of health care providers on migrants in Gauteng public health facilities, South Africa.* On behalf of my co-authors, this letter details the responses to the reviewers’ comments.

| **REVIEWER COMMENT** | **AUTHORS’ RESPONSE** | **PAGE REFERENCE** |
| --- | --- | --- |
| **GENERAL COMMENTS** | | |
| As the manuscript was submitted to the Call for Papers on Health Disparities and Inequities, Guest Editor Diana Burgess also assessed it and considered it for inclusion in the Collection. Please find her comments below, and consider them in your revision:  "I think it manuscript is suitable for inclusion. I also think the author was very responsive to the reviewer concerns. I had some minor concerns with the discussion section but these do not diminish my enthusiasm for the manuscript. The discussion presents specific results (e.g., "Health care providers obtained a score of 3.4 for the item on sensitivity to the health needs of migrants; and a score of 6.1 for providing the same quality of care"), rather than providing a broader interpretation of their findings. I also would like the authors to provide potential explanations for the effects of provider age on their attitudes and beliefs about migrants, since the effects of age were not clear cut and not consistent across items. Overall, I think this manuscripts makes a nice contribution to the literature and fills a gap in research about healthcare for migrant individuals.” | We have revised the discussion extensively and provided an explanation of the findings on sensitivity and quality. We have also revised the paragraph on provider age and discriminatory views. | p.22-23; p.24 |
| Reviewer #3: The paper provides significant evidence about the potentially discriminatory views of health-care providers in South Africa. However, although the authors have addressed the comments of reviewers to a large extent, the revised manuscript fails to address the concept of social exclusion in health, in a coherent way. | We have revised the introduction, and included a separate sub-section in the methods to explain the concept of social exclusion as used in this study. | p.4-5 |
| Health care provision varies across countries and time periods. It is unclear whether the paper intends to make a comparison of the South-African case with other European or non-European countries. Therefore, the examples of Canada and Greece are not very helpful in documenting the disparities in health care for migrant populations. | This paper does not intend to make a direct comparison between the South African case and other European/ non-European countries. Instead, we draw on health care providers’ perceptions and views on migrants in other host countries, as the only other examples in this literature. This comparison has been clarified in the text. | p.20 |
| The literature on social exclusion has to be updated. The authors may have a look at a newer report by WHO (World Health Organization Regional Office for Europe. Poverty and Social Exclusion in the WHO European Region: Briefing on policy issues produced through the WHO/European Commission equity project: World Health Organization Regional Office for Europe. 2010) | We have read the suggested references and updated the literature review.  The WHO report is 10 years old and focuses on the European region, which the reviewer questioned as a relevant comparison. |  |
| Relevant bibliography on social exclusion (i.e O’Donnell, P., O’Donovan, D. & Elmusharaf, K. Measuring social exclusion in healthcare settings: a scoping review. Int J Equity Health 17, 15 (2018), or the work of MIPEX health strand consortium , Ingleby et. Al. European Journal of Public Health, Volume 29, Issue 3, June 2019, Pages 458–462. | We have incorporated the O’Donnell article into the paper. However, the scoping review focused on measurement tools with the authors proposing that tools be developed for the primary health care setting. Many of the studies in the scoping review focus on mental health care. Our study used proxies of social exclusion as a relational concept and examined health care providers’ perspectives in hospital and PHC settings. | pp.5-6 |
| There are also more recent studies addressing the health inequalities in Greece (e.g. Health inequalities among migrant and native-born populations in Greece in times of crisis: the MIGHEAL study European Journal of Public Health, Volume 28, Issue suppl_5, December 2018, Pages 5–19, https://doi.org/10.1093/eurpub/cky225 | In this paper, we look at health care providers and their views on migrants. We do not focus on the broad and extensive literature on health inequalities, which is neither the focus nor within the scope of the current study. | N/A |
| Minor corrections: in p. 3, l. 7 it is unclear where the “conservative” refers to. | We have removed the word “conservative” from the text. | p.3 |
| In addition, in p. 5 lines 107-109 where “all these challenges” refer to. The manuscript needs language editing. | “All these challenges” refer to the health system challenges we allude to in the previous sentence. We have revised the phrase to read “All these health system challenges” for better coherence. | p.5 |
| The manuscript needs language editing. | We have undertaken an extensive edit and checking of the manuscript. |  |

Please do not hesitate to contact me should additional information be required.

Yours sincerely

**Ms Janine White**

**Corresponding author**

**Lecturer: Wits School of Public Health**
